# An Endophytic *Diaporthe apiculatum* Produces Monoterpenes with Inhibitory Activity against Phytopathogenic Fungi

**DOI:** 10.3390/antibiotics8040231

**Published:** 2019-11-22

**Authors:** Xiao-Yu Song, Huihua Wang, Fei Ren, Kaiying Wang, Guiming Dou, Xing Lv, Dong-Hui Yan, Gary Strobel

**Affiliations:** 1 Research Institute of Forest Ecology, Environment and Protection, Chinese Academy of Forestry, Key Open Laboratory of Forest Protection of National Forestry and Grassland Administration, Beijing 100091, China; songxiaoyucaf@gmail.com (X.-Y.S.); dougm@caf.ac.cn (G.D.); lvxing_15707483424@163.com (X.L.); 2 Department of Food and Biological Engineering, Beijing Vocational College of Agriculture, Beijing 102442, China; wnzy2012@163.com; 3 Experimental Center of Forestry in North China, Chinese Academy of Forestry, Beijing 102300, China; feifei3080@163.com; 4 Department of Plant Sciences, Montana State University, Bozeman, MT 59717, USA; uplgs@montana.edu

**Keywords:** endophytic fungus, *Diaporthe apiculatum*, *Leucaena leucocephala*, antifungal activity, inhibition, monoterpenes, (-)-4-terpineol

## Abstract

Volatile organic compounds (VOCs) from endophytic fungi are becoming a potential antibiotic resource. The inhibitive effects of VOCs produced by an endophytic fungus in *Leucaena leucocephala* were investigated on plant pathogens in this study. Using standard morphological methods and multigene phylogeny, the fungus was identified as *Diaporthe apiculatum* strain FPYF 3052. Utilizing a two- compartment Petri plate bioassay method, the VOCs from this fungus showed bioactivity ranging from 23.8% to 66.7% inhibition on eight plant pathogens within 24 hours. The SPME-GC/MS technique identified fifteen volatile compounds with dominant terpenoids γ-terpinene (39.8%), α-terpinene (17.2%), and (-)-4-terpineol (8.4%) from the VOCs. Commercial α-terpinene, γ-terpinene, and (-)-4-terpineol demonstrated inhibition on the tested pathogens at concentrations from 0.2 to 1.0 µl/ml within 72 h in the bioassay system. The inhibition rates were from 28% to 100% percent using 1.0 µl/ml within 48 h. (-)-4-Terpineol was the most active of the terpenoids causing up to 100% inhibition. The data illustrate that these monoterpenes play an important role in the inhibitive bioactivity of the VOCs of *D. apiculatum* FPYF 3052. Most importantly, (-)-4-terpineol is now for the first time, reported to have capability of strong antifungal activity and could be developed as an antibiotic substance.

## 1. Introduction

The white popinac tree, *Leucaena leucocephala,* has multiple purposes, among them being as a highly nutritious forage, animal food, medicines, and drought remediation [1,2]. Some tissues of *L. leucocephala* are able to produce bioactive metabolites conferring to defense or protection to the tree from environment stresses and attack by pathogens [3]. The seed oil of *L. leucocephala* was reported to have a concentration-dependent activity against both Gram-positive and Gram-negative bacteria [4]. The roots and leaf extracts of *L. leucocephala* have also demonstrated antimicrobial and antifungal activities [5]. At present, there is some evidence to show that endophytes could have a potential function in inhibiting harmful pests, like *Aspergillus fumigatus*, *Botrytis cinerea*, and *Mycobacterium tuberculosis* [6,7,8]. However, more endophytes of *L. leucocephala* need to be isolated and studied for their diversity and function in disease protection since only the ones studied so far are linked to nitrogen fixation, mycorrhizal associations, along with some seed-associated fungi [9,10].

Fungi from the genus *Diaporthe*, asexual genus *Phomopsis*, are one of the most common endophytic fungal communities found in plants [11], although many also were reported as pathogens [12]. The *Diaporthe* spp. are considered to be a potential source of metabolites that can be used in a variety of applications [13]. In addition, volatile organic compounds (VOCs) of endophytic fungi are being prospected to become a unique venue of non-toxic or less harmful applications for the biocontrol of pests. The VOCs are also being examined as to their roles in the defense and inhibitive effects against pathogens and insects in plants [6,7]. Those endophytic strains of *Diaporthe* genus isolated from some plants or trees, such as *Picrorhiza kurro* [14], *Odontoglossum* sp. [15], and *Catharanthus roseus* [7,16], are able to produce a unique mixture of inhibitive bioactive VOCs against many important fungal pathogens associated with crops and trees. The alcohols and terpenes are dominant components of VOCs in some fungal strains from the genus [15,17]. Especially terpenoids are reported as major components in *Diaporthe* spp. [7,17]. Some terpenoids produced by *Diaporthe* spp. show antifungal abilities and insect resistance in vitro experiments [18,19]. However, no knowledge on endophytic fungi form *Diaporthe* genus and their VOCs have been disclosed from *L. leucocephala* [1,20,21,22]. Therefore, we conducted an investigation into the antifungal activity of the VOCs produced by endophytic *Diaporthe apiculatum* strain FPYF 3052 isolated from wild *L. leucocephala* in Hainan, China. The bioactive constituents in the VOCs of *D. apiculatum* strain FPYF 3052 were determined, and the function of the active ingredients was confirmed. For the first time, the chief antifungal component of the VOCs was determined to be (-)-4-terpineol.

## 2. Results

### 2.1. The Identification on an Endophytic Isolate within the Diaporthe Genus

**Figure 1:**
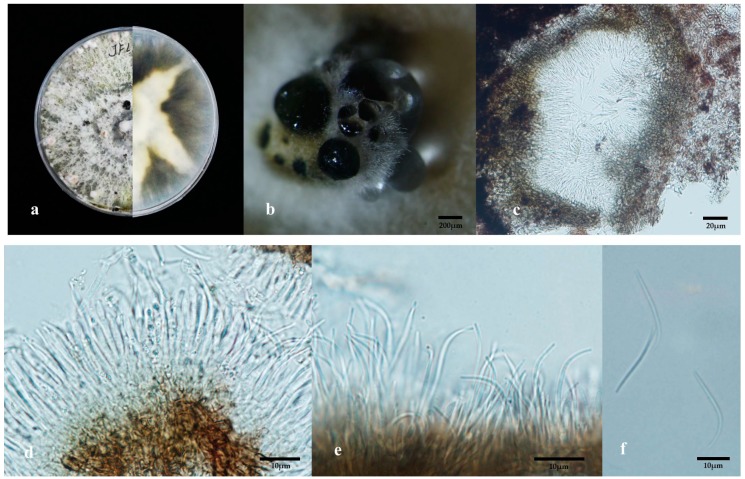
Morphological characteristics of the cultured *Diaporthe apiculatum* strain FPYF 3052 in 30 days culture. (**a**) *Diaporthe apiculatum* strain FPYF 3052 colony; (**b**) Conidiomata pycnidia; (**c**) Transverse section through conidiomata, showing conidiomata wall; (**d**,**e**) Conidiogenous cells; (**f**) Beta conidia. Cultural condition: grown in PDA media for 30 days at 25 °C in the dark.

The endophytic isolate formed a cyan-white compact mycelia colony with crenate margins after several days, it then developed into a colony having a moderate aerial mycelium, with a dirty white surface patched with pale olivaceous-grey on PDA at 25 °C for 1 week in the dark. It was able to secrete celadon yellow pigmentation in the center which was obvious as observed from the underside of the plate at 30 days of growth (Figure 1a). Its conidiomata pycnidia formed slowly and appeared only after 30 days (Figure 1b). The conidiomata occurred as globose, and were up to 400 μm in diameter, scattered or aggregated, brown to black, at conditions of 12 /12 hours alternative darkness and light at 25 °C for 4 weeks (Figure 1b). The walls of the stroma consisted of 3–6 layers with a brown texture (Figure 1c). Conidiogenous cells were 20–30 × 1.5–2 μm, cylindrical, with slight taper towards the apex, with visible periclinal thickening (Figure 1d,e). The beta conidia spores existed, and were subcylindrical, smooth, hyaline, rarely branched, apex bluntly rounded, curved, tapering towards the apex, 18–28 × 1.0–1.5 μm (Figure 1f). Alpha conidia and gamma conidia were absent. The isolate had at a growth rate of 8.3 mm/day in PDA at 25 °C in the dark.

**Figure 2:**
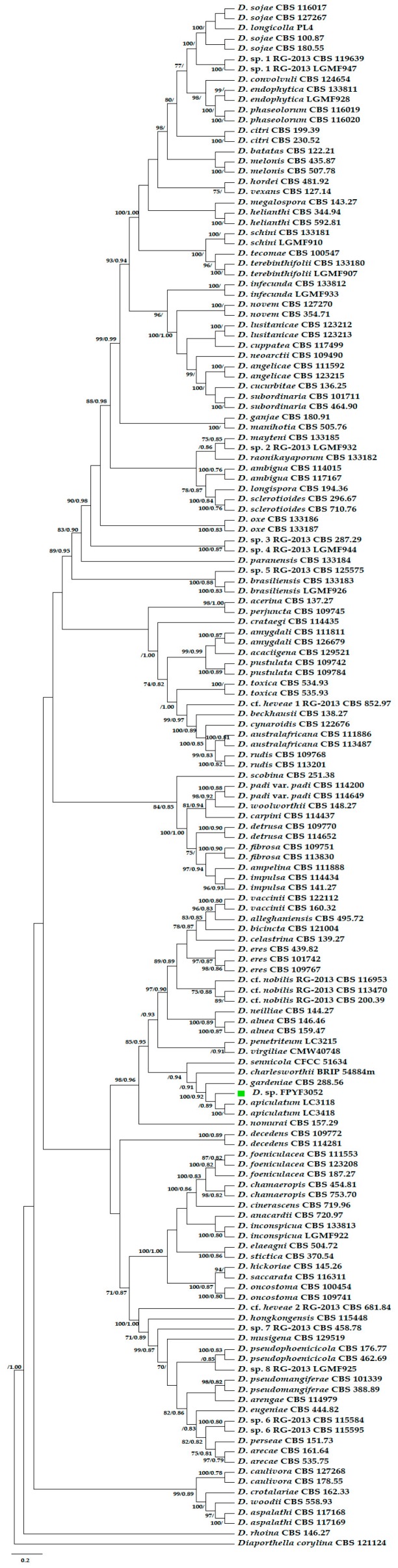
A multigene phylogenetic tree of ITS, *CAL, HIS, TEF1*, and *TUB* sequence alignment based on Bayes and MP analysis. The MP bootstrap values ≥70 or Bayesian probabilities PP ≥0.75 are marked above the branches (MP bootstrap value / Bayes poster probability value) The tree was rooted with *Diaporthella corylina*. The endophytic isolate was named with strain ID FPYF 3052 marked green box.

The isolate FPYF 3052 best fit the genus *Diaporthe* by a BLASTn search in the National Center for Biotechnology Information (NCBI) website with its sequenced five loci of ITS, *CAL, HIS, TEF1*, and *TUB*. The five-locus phylogenetic analysis with MP and Bayes inferences clustered the endophytic fungal strain FPYF 3052 in a clade with *D. apiculatum* with strong support of PP 0.89, and obviously distinguished it from other species (Figure 2). After comparing with morphological features of the ex-holotype strain, *D. apiculatum* LC3418, the present endophytic fungus was determined as a strain of the species *Diaporthe apiculatum.*

### 2.2. The VOCs’ Bioactivities of the Diaporthe apiculatum Strain FPYF 3052 against Plant Fungal Pathogens

The *D. apiculatum* strain FPYF 3052 was tested on the growth inhibition of eight selected fungal pathogens and two endophytic fungi with its volatile compounds (Table 1). These pathogenic fungi, *Alternaria alternata*, *Botryosphaeria dothidea*, *Botrytis cinerea*, *Cercospora asparagi*, *Colletotrichum gloeosporioides*, *Fusarium graminearum*, *Sphaeropsis sapinea*, and *Valsa sordida*, are important causal pathogenic agents to major trees such as poplars and pines, or agricultural crops. The FPYF 3052 showed various inhibitory bioactivities depending on pathogens and the tested timepoints. Firstly, all pathogens except *V. sordida* were inhibited by more than 12.20% during 72 hours of incubation. Furthermore, within 24 hours, the pathogens *B. cinerea* cfcc 83931 and *C. asparagi* were the most sensitive to the VOCs and were more than 56% growth inhibited. On the other hand, the pathogens *B. dothidea* and *A. alternata* were also highly sensitive to VOCs in being more than 36% inhibited. *V. sordida* was the least sensitive to the VOCs with the inhibition percentage around 3%. In addition, at 72 hours, the inhibitive intensity of the FPYF 3052 VOCs on the growth of pathogens decreased except *B. dothidea* compared to that within 24 hours. The maximum decline of 44% in inhibition rates occurred on *C. asparagi* from 24 to 72 hours. The two endophytic fungi *Annulohypoxylon* sp. FPYF3050 and *Gliocladium roseum* tested were insensitive to the VOCs.

### 2.3. The Qualification on VOCs of Endophytic Diaporthe apiculatum Strain FPYF 3052

The VOCs produced by *D. apiculatum* strain FPYF 3052 were identified by the SPME-GC/MS technique and fifteen VOC components were identified (Table 2). These identified compounds were clustered mainly into terpenoids, benzenes and benzene derivatives, alcohols, and hydrocarbons (Table S1). Terpenoid chemicals in the VOCs were abundant, accounting for 90.0% of the relative area (RA). Monoterpenes in the terpenoid group were dominant and most diverse including γ-terpinene, α-terpinene, α-thujene, β-phellandrene, and α-terpinol with RAs from 4.3% to 39.8%. The monoterpene alcohol, (-)-4-terpineol was also abundant with a relative area over 8.4%. The other three terpenoids were sesquiterpenes β-sesquiphellandrene, α-murolene, and (-)-α-himachalene. Other minor compounds found in the VOCs were 4,5-di-epi-aristolochene, Biphenylene,1,2,3,6,7,8,8a,8b-octahydro-4,5-dimethyl-, p-Cymene, and 2-cyclohexen-1-ol,1-methyl-4-(1-methylethyl)-,trans- with small RAs.

### 2.4. Bioactivities of Artificial Compounds and their Mixture on Plant Pathogens

The three monoterpenes including α-terpinene, γ-terpinene, (-)-4-terpineol were the most dominant components in the natural VOCs of the strain FPYF 3052. Therefore, their bioactivities contributing to pathogenic inhibitive effects were investigated with corresponding commercially available compounds. The results are displayed in Table 3, Table 4 and Table 5 using five treatment concentrations at 72 hours of incubation.

The commercial α-terpinene inhibited the growth of all tested pathogens in five concentrations from 0.2 to 1.0 μL/mL within 72 hours except on *C. asparagi* in 0.2 μL/mL in 72 hours (Table 3). The inhibitory effect intensity on the pathogens increased with increasing α-terpinene concentrations in each time tested. In 24 hours, the pathogen *S. sapinea* achieved the least inhibitory effect with 16.4% ± 4.4% inhibition rate and the pathogen *B. dothidea* achieved the strongest inhibitory effect with a 64.8% ± 0.5% inhibition rate at the concentration of 0.2 μL/mL. The inhibition rates on the two pathogens increased to 57.0% ± 1.4% and 100% with the concentration up to 1.0 μL/mL, respectively. The inhibition intensity of α-terpinene decreased along with increasing tested time from 24 to 72 hours to every pathogen in each treatment concentrations. In 0.2 μL/mL, the lowest inhibitive effect occurred on the pathogen *C. asparagi* in 48 hours with 4.9% ± 1.6% and even in 72 hours with no inhibitive effect with −5.1% ± 2.4% inhibition rates. However, the inhibition rate on the pathogen was 41.7% ± 3.0% in 24 hours. α-terpinene at the concentration of 1.0 μL/mL also exerted full inhibition on the growth of *F. graminearum* with 100% inhibitive rates in 24 hours besides of *B. dothidea*, and also exerted very strong inhibitive effects on the pathogens of *B. cinerea*, *C. asparagi*, and *V. sordida* with more than 90% inhibitive rates in 24 hours. The pathogen *C. gloeosporioides* was the least sensitive to the monoterpene inhibitory effect throughout all treatments with the lowest 9.8% inhibition rate at 0.2 μL/mL concentration in 24 hours and the highest 51.1% inhibition rate at 1 μL/mL concentration in 72 hours.

The effects of commercial γ-terpinene on the tested pathogenic fungi were recorded in Table 4. On all pathogens tested, the rates of inhibition ranged from 100% to the minimum inhibition rate of 3.7 % ± 1.4% in five concentrations from 0.2 to 1.0 μL/mL within 72 hours except on *C. asparagi* in 0.2 μL/mL in 72 hours. 

The effect intensity also showed an increasing increment with increasing concentrations to all tested pathogens within 72 hours. For instance, at 24 hours, the pathogen *B. cinerea* appeared to be the least inhibited at 26.6% ± 2.7% and the pathogen *B. dothidea* achieved the strongest inhibitory effect at 85.9% ± 0.5% at the concentration of 0.2 μL/mL. With the concentration increment to 1.0 μL/mL, the inhibitory effects on the two pathogens increased to 84.5% ± 1.0% and 98.0% ± 0.6%, respectively. The inhibition intensity of γ-terpinene on all pathogens decreased from 24 to 72 hours in all concentrations. For instance, at the concentration of 0.2 μL/mL, the inhibitive effect on the pathogen *F. graminearum* dropped from 63.3% ± 2.4% in 24 hours to only 3.7% ± 1.4% rate at 72 hours. 

Within 48 hours, γ-terpinene exerted an obvious inhibitory effect on all pathogens with more than 29% inhibition percentage in more than 0.4 μL/mL concentrations. The pathogen *C. gloeosporioides* performed generally the least sensitive to γ-terpinene with the lowest 9.0% inhibition rate at 0.2 μL/mL concentration in 72 hours and the highest 62.0% inhibition rate at 1 μL/mL concentration in 24 hours throughout the tested treatments. *F. graminearum* was the most sensitive pathogen to γ-terpinene effect. The pathogen initially was 100% inhibited at 0.4 μL/mL in 24 hours but was still high at 85.6% with 1.0 μL/mL to 72 hours. However, γ-terpinene stimulated growth of *C. asparagi* with the concentration of 0.2 μL/mL in 72 hours.

(-)-4-terpineol had inhibitive effects on the growths of the eight selected pathogens in the five concentrations from 0.2 to 1.0 μL/mL throughout 72 hours (Table 5). The effect presented with inhibition rates of pathogenic growths distributed from 9.8% to 97.3% depending on pathogens, treatment times, and (-)-4-terpineol concentrations. In 24 hours, at the concentration of 1 μL/mL of the maximum dose, *B. cinerea* showed highly sensitive performance with inhibition rate of 97.3%. In the minimum dose of (-)-4-terpineol (0.2 μL/mL), *S. sapinea* had the least insensitive performance, showing inhibition rate of 9.8%. The inhibitive intensity of (-)-4-terpineol on growths of all pathogens was mostly increased throughout 72 hours. For example, in 1.0 μL/mL concentration, the inhibition rate of (-)-4-terpineol against *F. graminearum* was 28.4% at 24 hours, 51.6% at 48 hours, and 69.3% at 72 hours. The four pathogens of *A. alternata*, *B. cinerea*, *C. asparagi*, and *F. graminearum* were completely inhibited to stop growing in exposure to 1.0 μL/mL over 5 days (data not showed). The IC_50_ values of (-)-4-terpineol inhibiting *A. alternata*, *B. cinerea*, *C. asparagi*, and *F. graminearum* were calculated to 0.49, 0.14, 1.09, and 0.33 μL/mL (Table 6), respectively.

The inhibitive effect of a mixture by α-terpinene, γ-terpinene, and (-)-4-terpineol on the plant pathogens was evaluated (Table 7). The mixture was deployed according to the ratio of the three compounds in natural FPYF 3052 VOCs (Table 2). The artificial mixture had visible inhibitive activity on all selected pathogens with the inhibition rate range from the lowest rate of 8.6% on *C. gloeosporioides* in 0.2 μL/mL in 72 hours to 100% inhibition on *B. dothidea* and *F. graminearum* in 1.0 μL/mL in 24 hours (Table 7). Most pathogens received their respective inhibitive effects near to maximum in 0.2 μL/mL within 48 hours. Therefore, the increment of the inhibitive effects on a pathogen was often not obvious after increasing the concentration as with individual chemicals (Table 3, 4, 5). However, the decreasing increments of the inhibitive effects on pathogens as a function of time were still clearly observed. Within 72 hours, the inhibition rates on the pathogen *B. dothidea* decreased from 99.3% ± 2.1% in 24 hours, 85.7% ± 6.3% in 48 hours to 38.6% ± 3.0% in 72 hours at 0.2 μL/mL concentration of the chemical mixture.

## 3. Discussion

Based on Bayes phylogenic inference (Figure 2,) as well as the colony and beta spore features, there was a highly similarity of our isolate to *D. apiculatum* strain LC3418 [23]. They both produced brownish yellow pigmentation in colony. Their beta conidia shared filiform, hyaline, tapering towards both apexes, hamate, or curved in appearance. Although the conidia of strain LC3418 was longer than that of our strain, this difference could exist among strains. Therefore, the native endophytic fungus from *L. leucocephala,* in this study, was ascribed as a strain of *Diaporthe apiculatum*. The ex-holotype of *D. apiculatum* strain LC3418 is also an endophytic fungus of *Camellia sinensis* [23]. However, further species determinants on more molecular loci and morphological phenotypes might be needed for this identification because it was not strongly supported by MP phylogenetic inference (Figure 2, Figure S1) and lacked alpha spores (Figure 1). The endophytic *Diaporthe*-like strain is the first reported from white popinac *L. leucocephala*.

This investigation showed that VOCs produced by the endophytic *D apiculatum* FPYF 3052 were able to inhibit the growth of fungal pathogens (Table 2). This result was consistent with evidence on the VOCs of some endophytic fungi of *Diaporthe* previously reported [7,14,15,17]. These fungi were recorded as *Diaporthe* sp. PR4 from the rhizome of *Picrorhiza kurroa* [14], *Diaporthe* sp. EC-4 from *Odontoglossum* sp. host in northern Ecuador [15], *Diaporthe* sp. Ut-1 from a plant of *Larrea tridentate* [17], and four *Diaporthe* spp. strains FPYF3053-3056 from *Catharanthus roseus* [7]. All of them also showed inhibition of various pathogens during different test times. The VOCs of *Diaporthe* sp. PR4 exhibited the greatest inhibition rate on *Rhizoctonia solani* by 100% after 24 hours [14]. The VOCs of *Diaporthe* sp. Ut-1 maximally reduced the radial growth of *Phytophthora palmivora* by 53.3% at 24 hours [17]. The *Diaporthe* sp. EC-4 VOCs appeared effective in the inhibition on growth of *Sclerotinia sclerotiorum* by 70.7% inhibition rate at 7 days [15]. *Diaporthe* spp. strains FPYF 3053-3056 exhibited the obvious inhibition rates against *A. alternata*, *B. cinerea*, *B. dothidea*, *C. asparagi*, *C. gloeosporioides*, *F. graminearum*, and *S. sapinea* between 8.9% and 50.4% during 48 hours [7]. The VOCs of *D. apiculatum* strain FPYF 3052 from *L. leucocephala* showed slightly stronger bioactivities with inhibition rates from 19.4% ± 1.8% to 51.9% ± 5.1% on the pathogens as compared to *Diaporthe* spp. FPYF 3053-3056 during the 48 hours test period (Table 1, [7]). This study added a new strain with antifungal activity of VOCs to the growing list of endophytic *Diaporthe* spp.

The components of VOCs of the *D. apiculatum* strain FPYF 3052 were significantly different than those VOCs from other *Diaporthe* spp. that have been reported. The VOCs by the FPYF 3052 was unique in that is was full of terpenes, especially with abundant and diverse monoterpenes (Table 2). The VOCs from *Diaporthe* sp. EC-4 was dominant with alcohol and contained only one monoterpene, *sabinene*, in a small amount [15]. *Diaporthe* sp. PR4 also produced VOCs with dominant alcohols and with rare monoterpenes limonene and phellandrene [14]. The VOCs of *Diaporthe* sp. Ut-1 contained no monoterpenes [17]. However, monoterpenoids made up the largest number of volatile compounds produced by FPYF 3052 accounting for 90.0% (Table 2). Although γ-terpinene and the other two monoterpenes, α-thujene and β-phellandrene, were the major constituents in VOCs of the *Diaporthe* spp. FPYF 3053, 3055-3056 [7], *Diaporthe apiculatum* FPYF 3052 VOCs had the dominant monoterpenes α-terpinene and (-)-4-terpineol besides γ-terpinene (Table 2). Furthermore, *Diaporthe apiculatum* FPYF 3052 did not produce α-thujene and β-phellandrene (Table 2). The three monoterpenes in VOCs of FPYF 3052 are well known as a constituents of the essential oils of many plants [18,19,21,24].

Monoterpenes contribute to the antibiotic activity in microbial volatiles [25]. In this study, artificial γ-terpinene, α-terpinene, (-)-4-terpineol, and their mixtures showed antifungal effect against tested pathogens (Table 3, Table 4, Table 5 and Table 7). The three volatile monoterpenes exhibited enhancing inhibitory effects on pathogens as a function of increased concentration. The inhibitory effects of γ-terpinene and α-terpinene on pathogens weakened as a function of increased time of exposure. This reason could be the adaption of the test fungus to the terpene. γ-Terpinene was considered to have volatile antifungal activities in the past [19,26,27]. It significantly affected some endophytic fungi singly or in mixtures with sabinene [19]. γ-Terpinene and p-cymene, which accounted for 1.18% of the total area (Table 3), are biosynthetically precursors of carvacrol and thymol which have high antifungal activity against food storage and phytopathogenic fungi [27]. It was also reported that there was a relationship between the strong antifungal activity and the high precursors (p-cymene and γ-terpinene) content in the oil of *Thymus numidicu* [28]. α-Terpinene was considered a component responsible for the trypanocidal effect in *Melaleuca alternifolia* essential oil with known efficacy in the treatment of trypanosomosis [18]. α-Terpinene enhanced the antibiotic activity of the essential oil from *Chenopodium ambrosioides* against *S. aureus* strains [29]. In this study, α-terpinene showed a direct inhibitive effect on the selected pathogenic fungi (Table 3).

Intriguingly, (-)-4-terpineol exerted an incremental inhibitive effect on pathogens as a function of time. It stopped the growth of *A. alternate*, *B. cinerea, C. asparagi*, and *F. graminearum* when they were exposed to the highest dose of (-)-4-terpineol over 5 days (Table 6). Furthermore, *B. cinerea* and *F. graminearum*, which were inhibited over five days, were not able to recover from growth inhibition even if (-)-4-terpineol was withdrawn (data not shown). Therefore, (-)-4-terpineol could be developed as a true gas antibiotic inhibitor. (-)-4-terpineol has not been reported as an antimicrobial volatile component from endophytes so far. It is similar chemically to terpene-4-alcohol (terpinen-4-ol), an isomer of (-)-4-terpineol, which has been reported as an antifungal and acts via disrupting cell walls, membranes, and cytoplasm, resulting in abnormal hyphae [30,31]. (-)-4-Terpineol may have similar antifungal mechanisms to terpene-4-alcohol. Future research is proposed to verify this hypothesis.

## 4. Materials and Methods 

### 4.1. Endophytic Fungal Isolation Identification

The endophytic fungus was isolated from healthy branches of *L. leucocephala*, growing in Jianfengling Mountain located in Sanya city of Hainan Province. The endophyte isolation process followed the procedure described previously [32]. Briefly, the surface of the branch sampling was washed by tap water and sterilized with 70% ethanol. Then, the tissue was sheared into several fragments with round 0.5 cm long size. The fragments were further sterilized with 75% ethanol for 60 s, 3% NaClO for 90 s, and washed in sterile water for 60 s three times. The sterilized fragments grew fungi on 2% water agar Petri plates at 25 °C in dark [32]. The PDA plate was wrapped with parafilm. The emerging mycelium tips from the plant tissue was transferred into PDA media (potato 250 g, sugar 20 g, agar 17 g, ddH_2_O 1000 mL). The fungus of interest aseptically dried barley seeds was stored in a freezer at −80 °C [8] in our laboratory with code FYFP3052 which can be provided by request.

For the fungal identification, the CTAB procedure was applied for retrieving the fungal genomic DNA from colonies growing on PDA for 7 days [32]. Five loci sequences were amplified from the genomic DNA by PCR. The five loci were calmodulin (*CAL*), histone H3 (*HIS*), ITS, translation elongation factor 1-alpha (*TEF1*), and beta-tubulin (*TUB*), and their correspondent regions amplified by primers CL1F/CL2A or CAL563F/CL2A [33], HISdiaF/HISdiaR [7], ITS1 and ITS4 [34], EF1-688F/EF1-1251R [35] and T1/Bt-2b or Bt2a/Bt-2b [36,37]. The sequences of the primers are listed in Table S2. For PCR amplification, the 25 µL volume system of PCR reaction mixtures were used following the Taq PCR MasterMix kits (Tiangen Biotech (Beijing) Co., Ltd.), including 2 µL (50–80 ng) of DNA template, 0.5 µL of each primer (10 µM), 12.5 µL of 2 × Taq PCR MasterMix (Tiangen Biotech (Beijing) Co., Ltd.) and 9.5 µL of double distilled water. The PCR cycling programs amplifying the five loci were similar, but annealing time and cycle number was adjusted for individual locus. The program was 94 °C for 5 min, 35 amplification cycles in 94 °C for 60 s, 55 °C for 30 s, and 72 °C for 1 min, the final extension step of 72 °C for 5 min for ITS amplification. The conditions were optimized for *CAL, TUB, TEF*, and *HIS* changed with a cycling program of 32 cycles and an annealing temperature at 55 °C for 60 s following the protocols [7]. The PCR products were cycle-sequenced with the BigDye® Terminator Cycle Sequencing Kit v. 3.1 (Applied Biosystems, Foster City, CA, USA) in an ABI Prism 3730 DNA Sequencer (Applied Biosystems, Foster City, CA, USA) at Biomed Company in Beijing. Then sequence data were assembled with forward and reverse sequences by BioEdit (ver. 7.2.0). The gene sequences were deposited in GenBank at NCBI (Accession numbers MH203053 for ITS, MH311618 for *TEF1*, MK554798 for *TUB*, MK554797 for *CAL*, MK554799 for *HIS*).

### 4.2. Phylogenetic Analysis on the FYFP 3052 within Diaporthe Genus

The evolutionary relationship of the strain FYFP 3052 within *Diaporthe* genus was inferred with Bayes and maximum parsimony (MP) phylogenetic analysis on a five-gene concatenated alignment of ITS, *TEF1, CAL, HIS*, and *TUB* regions. The correspondent sequences of reference taxa of *Diaporthe* species were downloaded from NCBI nucleotide databases (Table S3). The reference *Diaporthe* spp. strains were determined according to defined species by Crous [38,39] and new species reported afterward [23]. Total 149 taxa including the native strain FYFP 3052 and a root outgroup species [33], *Diaporthella corylina*, were applied for phylogenetic analysis. The MAFFTv.7 online program was used to sequences alignment with default parameters [40]. The five gene regions alignment was concatenated with SequenceMatrix [41]. Partition homogeneity on the concatenated alignment implemented in MyBayes (ver 3.1.2) determined if the five sequences data could be combined. The best evolutionary model on the concatenated sequences was performed by PartitionFinder 2.1.1 [42]. The evolutionary model GTR was applied to estimate phylogeny with a bootstrap support of 1000 replicates for maximum likelihood, respectively. Evidence on the trees were visualized and edited by Adobe Illustrator (Ver. 21.0.0.223), FigTree (Ver. 1.4.0) and TreeGraph 2 [43]. The reference sequences used to construct the phylogenetic tree were listed in Table 1 with their GenBank accession numbers.

### 4.3. Morphology

The strain FPYF 3052 was incubated on PDA petri wrapped with parafilm at 25 °C in the dark and growth rates were measured daily for 5 days. To induce sporulation, the isolate was inoculated on PDA with 12/12 hours alternative darkness and light at 25 °C [44]. Cultures were examined per 24 hours for the development of conidiomata. Conidia were taken from pycnidia and mounted in sterilized water. The shape and size of microscopic structures were observed and noted using a light microscope. At least five conidiomata, 30 conidiophores, conidia were measured to calculate the mean size and standard deviation (SD).

### 4.4. Fungal VOCs Inhibitive bioactivity on Plant Pathogens

The antagonism bioassay was performed in PDA plate within a 90 mm Petri referring to the tests in the reports previously [32,33,37]. A PDA medium in a Petri removed a 2 cm wide strip from the mid-portion, creating two isolated halves of PDA plate as a two-compartment Petri dish. The strain FYFP3052 was inoculated onto one semi-circular agar piece and incubated at 25 °C for 5 days for production of volatile compounds before the antagonism bioassay. The test pathogen of a 0.5 cm-diameter inoculum fetched from a 3–7 days-old culture was inoculated onto the opposite part agar piece. The Petri with PDA plate was wrapped with parafilm and incubated at 25 °C in dark for 72 hours. The growth of tested filamentous pathogens was measured at 24, 48, and 72 hours described previously [15,17]. The colony diameter of the test pathogens in a Petri was recorded in an average of four diameters disregarding the initial inoculum size. Inhibitive percentage on growth of a tested pathogen by bioactive VOCs of FYFP3052 was calculated as the formula:

| (a – b)/a | × 100%,(1)

a = mycelial colony diameter in control PDA plate Petri; 

b = mycelial colony diameter in the PDA plate Petri plate with the treatment.

The test plant pathogens were Alternaria alternata cfcc 82113, Botryosphaeria dothidea cfcc 87875, Botrytis cinerea cfcc 83931, Cercospora asparagi hmh-3-1, Colletotrichum gloeosporioides cfcc 86446, Fusarium graminearum cfcc 50512, Sphaeropsis sapinea cfcc 88430, and Valsa sordida cfcc 84641, which are very important pathogens on crops, fruits, vegetables, and trees. The strains with the abbreviation of cfcc were from China Forestry Culture Collection Center. Other strains were from our laboratory. The test plant endophytic fungi were Annulohypoxylon sp. FPYF3050 [32] and Gliocladium roseum TGL 18-2f-1-3 isolated from seeds of Catharanthus roseus, which is an endophytic fungus [45]. All tests were made in at least five replicates. Control cultures were obtained by growing each plant pathogen alone under the same conditions.

### 4.5. The Endophytic Fungal Volatile Metabolites

The cultures of the FYFP3052 strain on PDA in 90 mm-diameter Petri in five days at 25 ± 2 °C were analyzed for volatile compounds which were applied for bioassay on test pathogens. The volatiles in the headspace of the Petri plate were adsorbed by a SPME fiber syringe of 50/30 divinylbenzene/carboxen on polydimethylsiloxane (Supelco, Bellefonte, PA, USA) for 40 min according to the fiber application manual and the published papers [1,2,3,6]. Prior to adsorption of the volatiles, the fiber was conditioned at 220 °C for 40 min, and a 0.5 mm-diameter hole by a drill was made for putting the fiber into for the adsorption [8]. All treatments and checks were done at least in triplicate.

The compounds were desorbed by inserting the fiber into the TRACE DSQ inlet (Thermo Electron Corporation, Beverly, MA, USA) at 240 °C, splitless mode. The desorbed compounds were separated on HP-5MS capillary column (30.0 m × 0.25 mm × 0.25 m) employing helium as carrier gas at a flow rate of 1 mL/ min. The oven temperature was 40 °C for 2 min, then up to 220 °C at 7 °C/min. Electronic ionization energy was 70 eV and the mass range scanned was 41 to 560 amu (atomic mass unit) at a scan rate of 5 spec/s. The transfer line and ionization chamber temperatures were 250 and 200 °C. The volatile compounds were tentatively identified via library comparison using the NIST 11 database (Scientific Instrument Services, Inc., Ringoes, NJ, USA) and all chemical compounds were described in this report following the NIST database chemical identity.

Tentative compound identity was based on at least a 70% quality match with the National Institute of Standards and Technology (NIST) database information for each compound. Data acquisition and data processing were performed with the Hewlett Packard ChemStation software system (Version 2.0, Scientific Instrument Services, Inc., Ringoes, NJ, USA). Relative amounts of individual components of the treatments were determined and expressed as percentages of the peak area within the total peak area and as an average of the three replicates. Additionally, available authentic standards (≥95% purity, Sigma Aldrich) were used for conclusive identification of the volatile compounds. The compounds contributed only from pure PDA medium were subtracted from the data analyses. Statistical significance (*p* < 0.01) was evaluated by analysis of variance (ANOVA) followed by the Tukey 5% test.

### 4.6. Bioactive Effect of Some Commercial Terpenoids Components on Plant Pathogens

Three components, γ-terpinene, α-terpinene, and (-)-4-terpineol, were determined as the most prominent compounds in natural VOCs from the FYPF 3052 according to pike relative area with GC-MS analysis (Table 2). Their corresponding commercial chemicals were obtained, γ-terpinene (Sigma Aldrich, ≥95%, GC), α-terpinene (Sigma Aldrich, ≥95%, GC), and (-)-4-terpineol (Sigma Aldrich, ≥95%, GC). And they were used to prepare a mixture containing the compounds in the same proportions as those determined by GC/MS analysis of the natural mixture.

The artificial compounds and their mixture were placed in a tightly sealed container (such as microcentrifuge tubes) and stored at 0 °C. A PDA medium in a Petri was divided into two nearly equal parts by removing a 2 cm wide strip in the center. A sterile plastic well (caps removed from 1.5mL microcentrifuge tubes) was placed in the center of one half PDA plate, whilst test plant-pathogenic micro-organisms (5 × 5 × 5 mm agar blocks of freshly growing test organisms) inoculated in the other half plate [8]. These compounds did not dissolve the plastic. Five different doses of the artificial compound mixture with 10, 20, 30, 40, 50 µL were added simultaneously to the well (the volume of the headspace is 50 mL). And the plate was immediately sealed with two layers of parafilm. Five plates for each dose were made; the control plates (one for each pathogen) did not receive VOCs in the micro-caps. The plates were incubated at 25 °C for one day and then the growth of the test organisms was measured after 24 hours of incubation.

IC_50_s of the artificial VOC compounds was assessed after 48 hours and compared to a control plate. Pathogens, which showed no growth after that period, were determined to be 100% inhibited. Those which showed no growth after 48 hours and no growth after inoculation onto PDA immediately following the 48 hours assessment were considered dead. The IC_50_ calculation was determined by dividing the amount of the artificial compounds required to cause 50% inhibition by the total air space in the Petri dish (50 mL) [8].

Statistical analysis: Each treatment and control were necessary to be at least triplicates in all experiment for this research. Species were exposed to five doses of each compound/mixture. In the control treatment isolates grew with no volatiles. All treatments had five replicates. 

Statistical significance (p < 0.01) was evaluated by analysis of variance (ANOVA) followed by the Tukey 5% test.

## 5. Conclusions

An endophytic fungal strain FPYF 3052 of *Diaporthe apiculatum* was obtained from its host white popinac, *L. leucocephala.* The strain was able to produce antibiotic VOCs to inhibit fungal plant pathogens via a unique set of abundant terpenoid compounds, especially dominant were the monoterpenes, γ-terpinene, α-terpinene, and (-)-4-terpineol. These monoterpenes, among other compounds, seem to play an important role in the fungal inhibition of its VOCs. Furthermore, we here note that the high content of monoterpenes in the *Diaporthe apiculatum* FPYF 3052 VOCs could make it an available source of potential biofuels and for other industrial and bio-pharmaceutical uses [17,32,46].

## Figures and Tables

**Table 1:** Growth inhibition percentages of plant pathogens with volatile organic compounds (VOCs) of *Diaporthe apiculatum* strain FPYF 3052 determined by two-compartment Petri plate bioassay method.

Pathogen/ Endophyte		24 h		48 h		72 h	
		Inhibition Percentage	P-Value	Inhibition Percentage	P-Value	Inhibition Percentage	P-Value
**Pathogen**	*A. alternata* cfcc 82113	36.8 ± 0.8	0.00	25.7 ± 1.1	0.00	20.1 ± 3.0	0.00
	*B. dothidea* cfcc 87875	47.2 ± 2.4	0.00	50.1 ± 2.4	0.00	NE *	
	*B. cinerea* cfcc 83931	66.7 ± 3.8	0.00	51.9 ± 5.1	0.00	32.1 ± 7.3	0.02
	*C. asparagi* hmh-3-1	56.3 ± 3.0	0.00	38.1 ± 2.8	0.00	12.2 ± 2.7	0.00
	*C. gloeosporioides* cfcc 86446	23.8 ± 3.9	0.00	19.4 ± 1.8	0.00	18.2 ± 0.8	0.00
	*F. graminearum* cfcc 50512	30.3 ± 2.0	0.01	26.8 ± 4.8	0.03	NE	
	*S. sapinea* cfcc 88430	27.4 ± 2.9	0.01	24.3 ± 0.5	0.00	NE	
	*V. sordida* cfcc 84641	3.8 ± 0.6	0.00	NE		NE	
**Endophyte**	*Annulohypoxylon* sp. FPYF3050	2.5 ± 0.5	0.02	0.7 ± 0.2	0.02	NE	
	*G. roseum* TGL 18-2f-1-3	-3.9 ± 1.2	0.04	3.8 ± 1.1	0.04	NE	

Note: Inhibition percentages were shown as the means of three measurements of diameters with standard deviation (*n* = 3). * No data due to colony growing full over petri plate.

**Table 2:** The components in VOCs of the endophytic fungus *Diaporthe apiculatum* strain FPYF 3052.

Retention Time (min)	Molecular Weight (AMU)	Compounds	Quality (%)	Abundance (Relative Area*)
7.65	136	α-thujene	89.4	7.9
8.79	136	β-phellandrene	87.7	4.6
9.87	136	α-terpinene	86.5	17.1
10.08	134	p-cymene	79.8	1.2
10.92	136	γ-terpinene	91.3	39.8
11.61	136	α-terpinolen	86.3	4.3
11.88	154	2-cyclohexen-1-ol, 1-methyl-4-(1-methyl ethyl)-, trans-	78.6	0.9
13.76	154	(-)-4-terpineol	88.2	8.4
14.12	352	Unknown **	64.7	0.4
18.41	188	Biphenylene,1,2,3,6,7,8,8a,8b-octahydro-4,5-dimethyl-	76.9	2.3
19.04	204	(-)-α-himachalene	77.9	0.7
19.91	204	4,5-di-epi-aristolochene	83.6	4.1
20.13	204	β-sesquiphellandrene	81.4	5.5
20.46	204	α-muurolene	83.9	1.7
28.46	272	Unknown	64.3	1.1

*: The relative area figure presents the percentage amount of each compound in total area relative to all listed compounds detected for one strain. **: Compounds assigned as unknown with lower than 70% quality match. The compounds in VOCs of control PDA plate were subtracted.

**Table 3:** Effects of α-terpinene on fungal pathogens of plants determined by two-compartment Petri plate bioassay method.

Pathogen	Day	0.2 μL/mL		0.4 μL/mL		0.6 μL/mL		0.8 μL/mL		1.0 μL/mL	
		Inhibition Percentage	P-Value	Inhibition Percentage	P-Value	Inhibition Percentage	P-Value	Inhibition Percentage	P-Value	Inhibition Percentage	P-Value
*A. alternata* cfcc 82113	24 h	45.7 ± 0.3	0.02	69.2 ± 4.7	0.01	73.4 ± 0.5	0.00	74.7 ± 1.8	0.00	74.3 ± 1.4	0.02
	48 h	20.3 ± 3.6	0.01	36.6 ± 5.5	0.03	55.4 ± 1.5	0.01	62.7 ± 3.9	0.00	70.9 ± 1.0	0.00
	72 h	NE *									
*B. dothidea* cfcc 87875	24 h	64.8 ± 0.5	0.00	85.6 ± 1.9	0.00	96.6 ± 0.8	0.00	98.5 ± 0.8	0.00	100.0 ± 0.0	0.00
	48 h	27.8 ± 1.0	0.00	42.8 ± 0.9	0.00	63.4 ± 0.6	0.00	71.9 ± 3.3	0.00	78.5 ± 1.4	0.00
	72 h	12.7 ± 0.6	0.00	20.3 ± 1.6	0.00	35.9 ± 0.4	0.00	41.4 ± 3.5	0.00	48.4 ± 2.3	0.00
*B. cinerea* cfcc 83931	24 h	23.7 ± 3.6	0.00	55.9 ± 1.2	0.00	73.8 ± 2.1	0.00	88.0 ± 0.1	0.00	90.9 ± 0.6	0.00
	48 h	6.0 ± 0.7	0.01	35.5 ± 4.6	0.00	55.9 ± 3.2	0.00	65.9 ± 1.0	0.00	74.1 ± 1.1	0.00
	72 h	NE									
*C. asparagi* hmh-3-1	24 h	41.7 ± 3.0	0.00	66.7 ± 5.3	0.00	77.8 ± 1.8	0.00	88.9 ± 0.9	0.00	93.3 ± 2.2	0.00
	48 h	4.9 ± 1.6	0.04	16.3 ± 1.6	0.00	16.6 ± 2.2	0.00	26.4 ± 0.9	0.00	28.9 ± 2.5	0.00
	72 h	-5.1 ± 2.4	0.02	1.2 ± 0.3	0.04	6.5 ± 2.8	0.04	8.2 ± 0.7	0.00	9.1 ± 1.6	0.00
*C. gloeosporioides* cfcc 86446	24 h	25.6 ± 1.4	0.00	34.2 ± 2.9	0.00	48.1 ± 4.3	0.00	52.8 ± 3.2	0.00	51.1 ± 2.0	0.00
	48 h	16.3 ± 0.5	0.00	23.3 ± 0.9	0.00	36.6 ± 3.4	0.00	39.8 ± 3.0	0.00	39.8 ± 2.2	0.00
	72 h	9.8 ± 1.0	0.00	16.3 ± 0.7	0.00	27.8 ± 2.6	0.00	33.3 ± 0.8	0.00	35.2 ± 0.7	0.00
*F. graminearum* cfcc 50512	24 h	44.6 ± 1.0	0.00	69.9 ± 4.3	0.00	87.5 ± 5.4	0.00	95.1 ± 0.6	0.00	100.0 ± 0.0	0.00
	48 h	16.4 ± 3.5	0.00	31.2 ± 0.4	0.04	59.0 ± 2.9	0.01	77.4 ± 1.1	0.00	89.3 ± 1.3	0.00
	72 h	NE									
*S. sapinea* cfcc 88430	24 h	16.4 ± 4.4	0.00	35.2 ± 2.7	0.00	45.7 ± 3.6	0.00	53.3 ± 4.0	0.00	57.0 ± 1.4	0.00
	48 h	15.4 ± 1.7	0.00	25.9 ± 1.4	0.00	36.9 ± 2.0	0.00	41.3 ± 1.2	0.00	50.7 ± 0.4	0.00
	72 h	9.5 ± 5.1	0.02	22.5 ± 3.6	0.00	29.9 ± 4.0	0.00	33.4 ± 1.9	0.00	35.5 ± 1.6	0.00
*V. sordida* cfcc 84641	24 h	52.0 ± 1.6	0.00	70.8 ± 1.6	0.00	89.2 ± 1.3	0.00	93.5 ± 1.1	0.00	97.8 ± 0.7	0.00
	48 h	NE	0.00	65.3 ± 2.2	0.00	74.1 ± 5.0	0.00	81.3 ± 3.1	0.00	84.9 ± 2.1	0.00
	72 h	NE									

* Stop value calculation for control of the pathogen growing over PDA plate in Petri.

**Table 4:** Effects of γ-terpinene on fungal pathogens of plants determined by two-compartment Petri plate bioassay method.

Pathogen	Day	0.2 μL/mL		0.4 μL/mL		0.6 μL/mL		0.8 μL/mL		1.0 μL/mL	
		Inhibition Percentage	P-Value	Inhibition Percentage	P-Value	Inhibition Percentage	P-Value	Inhibition Percentage	P-Value	Inhibition Percentage	P-Value
*A. alternata* cfcc 82113	24 h	48.7 ± 4.3	0.00	69.6 ± 3.5	0.00	73.0 ± 4.0	0.00	73.9 ± 4.8	0.00	78.4 ± 1.6	0.00
	48 h	24.7 ± 2.5	0.02	59.2 ± 2.6	0.00	65.5 ± 1.5	0.00	66.7 ± 2.8	0.00	68.2 ± 1.9	0.00
	72 h	NE *									
*B. dothidea* cfcc 87875	24 h	85.9 ± 0.5	0.00	95.8 ± 1.0	0.00	94.6 ± 0.8	0.00	97.5 ± 0.7	0.00	98.0 ± 0.6	0.00
	48 h	45.9 ± 4.7	0.00	82.1 ± 4.1	0.00	87.3 ± 2.8	0.00	94.6 ± 2.0	0.00	94.7 ± 0.6	0.00
	72 h	NE	0.00	NE	0.00	74.1 ± 5.0	0.00	81.3 ± 3.1	0.00	84.9 ± 2.1	0.00
*B. cinerea* cfcc 83931	24 h	26.6 ± 2.7	0.00	80.2 ± 1.4	0.00	83.0 ± 0.6	0.00	84.3 ± 0.3	0.00	84.5 ± 1.0	0.00
	48 h	7.2 ± 1.5	0.00	39.5 ± 3.7	0.00	70.3 ± 3.2	0.00	80.1 ± 0.9	0.00	79.7 ± 0.3	0.00
	72 h	NE									
*C. asparagi* hmh-3-1	24 h	72.1 ± 2.4	0.00	81.4 ± 1.6	0.00	90.7 ± 0.8	0.00	100.0 ± 0.0	0.00	100.0 ± 0.0	0.00
	48 h	25.6 ± 5.3	0.00	47.6 ± 3.5	0.00	45.1 ± 3.4	0.00	47.6 ± 1.9	0.00	70.7 ± 0.8	0.00
	72 h	-9.3 ± 4.4	0.02	2.8 ± 1.1	0.01	15.0 ± 1.5	0.00	22.5 ± 7.3	0.00	31.9 ± 4.6	0.00
*C. gloeosporioides* cfcc 86446	24 h	46.4 ± 6.3	0.00	48.7 ± 3.3	0.00	51.5 ± 4.2	0.01	54.9 ± 2.4	0.00	62.0 ± 1.5	0.00
	48 h	19.2 ± 4.5	0.02	29.5 ± 1.5	0.00	35.5 ± 4.8	0.01	38.3 ± 2.7	0.00	38.6 ± 2.1	0.00
	72 h	9.0 ± 0.8	0.00	15.8 ± 0.5	0.00	25.8 ± 1.0	0.00	30.9 ± 0.9	0.00	31.7 ± 0.2	0.00
*F. graminearum* cfcc 50512	24 h	63.3 ± 2.4	0.00	100.0 ± 0.0	0.00	100.0 ± 0.0	0.00	100.0 ± 0.0	0.00	100.0 ± 0.0	0.00
	48 h	13.0 ± 3.1	0.02	73.7 ± 1.0	0.00	89.3 ± 6.2	0.00	95.4 ± 0.2	0.00	100.0 ± 0.0	0.00
	72 h	3.7 ± 1.4	0.04	43.9 ± 2.6	0.00	69.6 ± 1.1	0.00	83.1 ± 0.4	0.00	85.6 ± 3.2	0.00
*S. sapinea* cfcc 88430	24 h	62.5 ± 5.3	0.00	67.7 ± 3.5	0.00	72.1 ± 1.1	0.00	71.4 ± 0.8	0.00	73.8 ± 3.3	0.00
	48 h	26.6 ± 2.6	0.00	47.3 ± 4.2	0.00	62.7 ± 1.0	0.00	64.5 ± 1.4	0.00	70.1 ± 1.7	0.00
	72 h	10.0 ± 4.4	0.01	30.3 ± 4.4	0.00	49.1 ± 3.8	0.00	60.1 ± 3.3	0.00	68.4 ± 1.1	0.00
*V. sordida* cfcc 84641	24 h	71.9 ± 2.6	0.00	85.9 ± 0.3	0.00	86.3 ± 3.4	0.00	86.4 ± 2.3	0.00	88.2 ± 3.4	0.00
	48 h	NE									
	72 h	NE									

* Stop value calculation for control of the pathogen growing over PDA plate in Petri.

**Table 5:** Effects of (-)-4-terpineol on fungal pathogens of plants determined by two-compartment Petri plate bioassay method.

Pathogen	Day	0.2 μL/mL		0.4 μL/mL		0.6 μL/mL		0.8 μL/mL		1.0 μL/mL	
		Inhibition Percentage	P-Value	Inhibition Percentage	P-Value	Inhibition Percentage	P-Value	Inhibition Percentage	P-Value	Inhibition Percentage	P-Value
*A. alternata* cfcc 82113	24 h	14.0 ± 4.6	0.01	20.0 ± 6.0	0.04	24.5 ± 4.9	0.00	26.6 ± 6.4	0.01	49.1 ± 0.3	0.00
	48 h	25.2 ± 4.7	0.00	27.4 ± 2.2	0.01	40.5 ± 5.6	0.00	53.5 ± 4.3	0.00	62.6 ± 3.3	0.00
	72 h	34.1 ± 5.3	0.00	37.3 ± 7.2	0.00	52.4 ± 5.9	0.00	64.7 ± 5.3	0.00	70.7 ± 3.0	0.00
*B. dothidea* cfcc 87875	24 h	40.4 ± 0.8	0.00	54.2 ± 3.3	0.00	62.0 ± 6.9	0.00	64.9 ± 1.9	0.00	71.4 ± 4.5	0.00
	48 h	54.2 ± 1.2	0.00	64.5 ± 2.0	0.00	70.8 ± 0.2	0.00	79.3 ± 0.8	0.01	85.0 ± 1.4	0.00
	72 h	NE^*^	0.00	65.3 ± 2.2	0.00	74.1 ± 5.0	0.00	81.3 ± 3.1	0.00	84.9 ± 2.1	0.00
*B. cinerea* cfcc 83931	24 h	52.0 ± 1.1	0.00	57.4 ± 0.0	0.00	61.5 ± 4.5	0.00	80.1 ± 2.8	0.00	97.3 ± 0.1	0.00
	48 h	63.3 ± 1.1	0.00	74.1 ± 0.4	0.00	76.8 ± 2.4	0.00	87.6 ± 1.6	0.00	96.9 ± 0.3	0.00
	72 h	NE									
*C. asparagi* hmh-3-1	24 h	18.0 ± 3.6	0.00	27.2 ± 1.8	0.01	28.0 ± 1.0	0.00	29.2 ± 4.7	0.01	34.4 ± 2.7	0.00
	48 h	16.4 ± 1.3	0.00	32.3 ± 2.1	0.00	33.6 ± 3.2	0.00	35.1 ± 4.3	0.01	38.8 ± 2.8	0.00
	72 h	29.3 ± 0.8	0.00	33.3 ± 4.9	0.00	40.3 ± 1.0	0.00	46.9 ± 2.4	0.01	49.9 ± 4.5	0.00
*C. gloeosporioides* cfcc 86446	24 h	14.3 ± 4.8	0.01	20.8 ± 5.1	0.00	19.7 ± 3.6	0.01	30.5 ± 1.2	0.00	27.5 ± 2.7	0.00
	48 h	25.7 ± 5.2	0.00	30.3 ± 3.9	0.00	28.4 ± 4.0	0.00	42.0 ± 4.2	0.00	37.5 ± 5.4	0.00
	72 h	28.7 ± 5.0	0.00	33.9 ± 1.9	0.00	33.6 ± 3.4.	0.00	48.2 ± 5.9	0.00	45.0 ± 4.3	0.00
*F. graminearum* cfcc 50512	24 h	18.5 ± 2.4	0.00	20.2 ± 1.4	0.00	22.7 ± 3.4	0.00	24.7 ± 4.2	0.00	28.4 ± 1.0	0.00
	48 h	39.5 ± 1.9	0.00	46.5 ± 5.3	0.00	49.3 ± 0.8	0.00	51.2 ± 0.5	0.00	51.6 ± 1.7	0.00
	72 h	46.9 ± 1.2	0.00	54.3 ± 2.2	0.00	58.9 ± 3.7	0.00	62.5 ± 4.4	0.00	69.3 ± 2.1	0.00
*S. sapinea* cfcc 88430	24 h	9.8 ± 1.5	0.00	10.4 ± 0.2	0.00	10.8 ± 0.8	0.00	11.2 ± 0.7	0.00	15.7 ± 1.5	0.00
	48 h	23.2 ± 3.9	0.00	26.1 ± 1.4	0.00	28.4 ± 0.6	0.00	31.7 ± 1.7	0.00	35.3 ± 2.6	0.00
	72 h	35.4 ± 4.8	0.00	36.9 ± 2.0	0.00	37.2 ± 4.0	0.00	45.3 ± 2.0	0.00	50.0 ± 1.8	0.00
*V. sordida* cfcc 84641	24 h	22.4 ± 4.7	0.00	27.5 ± 2.9	0.00	29.7 ± 0.8	0.00	27.3 ± 2.3	0.00	31.5 ± 4.6	0.00
	48 h	28.3 ± 4.1	0.00	37.5 ± 4.4	0.00	40.9 ± 3.3	0.00	46.8 ± 2.6	0.00	52.7 ± 3.9	0.00
	72 h	NE									

* Stop value calculation for control of the pathogen growing over PDA plate in Petri.

**Table 6:** IC_50_ of commercial (-)-4-terpineol on fungal pathogens of plants.

Pathogen	Minimum vol. to Cause 100% Inhibition (μL)	IC_50_ of (-)-4-terpineol (μL/ mL)
*A. alternata* cfcc 82113	40	0.49 ± 0.09
*B. dothidea* cfcc 87875	N/O	N/O
*B. cinerea* cfcc 83931	40	0.14 ± 0.03
*C. asparagi* hmh-3-1	40	1.09 ± 0.10
*C. gloeosporioides* cfcc 86446	N/O	N/O
*F. graminearum* cfcc 50512	40	0.33 ± 0.05
*S. sapinea* cfcc 88430	N/O	N/O
*V. sordida* cfcc 84641	N/O	N/O

N/O: no value for fungal continuingly growing in the test.

**Table 7:** Effects of mixtures (α-terpinene:γ-terpinene:(-)-4-terpineol=26:61:13) on fungal pathogens of plants determined by two-compartment Petri plate bioassay method.

Pathogen	Day	0.2 μL/mL		0.4 μL/mL		0.6 μL/mL		0.8 μL/mL		1.0 μL/mL	
		Inhibition Percentage	P-Value	Inhibition Percentage	P-Value	Inhibition Percentage	P-Value	Inhibition Percentage	P-Value	Inhibition Percentage	P-Value
*A. alternata* cfcc 82113	24 h	65.9 ± 0.4	0.01	80.0 ± 3.7	0.00	83.5 ± 2.9	0.01	80.0 ± 3.7	0.00	81.7 ± 3.9	0.01
	48 h	41.3 ± 3.8	0.00	60.9 ± 2.5	0.00	70.9 ± 1.3	0.00	77.7 ± 1.1	0.01	74.0 ± 2.2	0.00
	72 h	NE									
*B. dothidea* cfcc 87875	24 h	99.3 ± 2.1	0.00	99.3 ± 0.2	0.00	99.5 ± 0.2	0.00	99.3 ± 0.4	0.00	100.0 ± 0.0	0.00
	48 h	85.7 ± 6.3	0.00	85.7 ± 2.7	0.00	93.8 ± 1.0	0.00	95.3 ± 2.2	0.00	99.2 ± 0.4	0.00
	72 h	38.6 ± 3.0	0.00	63.4 ± 2.3	0.00	73.6 ± 2.8	0.00	89.4 ± 3.5	0.00	97.6 ± 1.5	0.00
*B. cinerea* cfcc 83931	24 h	87.0 ± 1.1	0.00	94.7 ± 0.8	0.01	96.3 ± 1.3	0.00	96.5 ± 1.9	0.00	97.4 ± 1.2	0.00
	48 h	61.2 ± 4.1	0.00	74.3 ± 2.8	0.00	82.0 ± 1.0	0.00	89.0 ± 1.8	0.00	92.8 ± 1.8	0.00
	72 h	NE									
*C. asparagi* hmh-3-1	24 h	92.8 ± 0.9	0.00	93.4 ± 1.1	0.00	92.1 ± 0.5	0.00	96.1 ± 1.2	0.00	98.0 ± 1.2	0.00
	48 h	26.8 ± 4.4	0.00	50.4 ± 2.0	0.00	62.3 ± 2.1	0.00	68.0 ± 4.0	0.00	67.4 ± 0.3	0.00
	72 h	10.6 ± 3.6	0.01	24.4 ± 2.2	0.00	32.3 ± 0.7	0.00	43.2 ± 1.4	0.00	50.8 ± 1.0	0.00
*C. gloeosporioides* cfcc 86446	24 h	36.0 ± 1.9	0.00	39.7 ± 2.1	0.00	45.1 ± 1.2	0.00	45.5 ± 5.6	0.00	48.2 ± 4.8	0.00
	48 h	23.7 ± 1.5	0.00	27.8 ± 1.3	0.00	33.5 ± 2.5	0.00	37.5 ± 3.0	0.00	40.1 ± 2.7	0.00
	72 h	8.6 ± 1.0	0.00	11.2 ± 2.2	0.00	24.3 ± 2.0	0.00	26.5 ± 4.2	0.00	31.8 ± 1.3	0.00
*F. graminearum* cfcc 50512	24 h	88.3 ± 0.7	0.00	99.5 ± 0.1	0.00	99.6 ± 0.1	0.00	100.0 ± 0.0	0.00	100.0 ± 0.0	0.00
	48 h	69.7 ± 3.6	0.01	88.0 ± 1.5	0.01	94.7 ± 2.4	0.00	96.7 ± 1.1	0.00	97.9 ± 1.7	0.00
	72 h	NE									
*S. sapinea* cfcc 88430	24 h	42.9 ± 1.4	0.00	54.0 ± 0.4	0.00	58.9 ± 1.9	0.00	62.1 ± 0.6	0.00	62.7 ± 0.4	0.00
	48 h	32.7 ± 1.6	0.00	49.7 ± 2.8	0.00	56.6 ± 1.0	0.00	57.1 ± 0.3	0.00	57.7 ± 2.4	0.00
	72 h	NE									
*V. sordida* cfcc 84641	24 h	84.9 ± 1.9	0.00	97.7 ± 0.6	0.00	98.5 ± 0.8	0.00	98.5 ± 1.0	0.00	98.9 ± 0.7	0.00
	48 h	48.2 ± 0.7	0.00	75.3 ± 6.1	0.00	84.7 ± 1.6	0.00	92.1 ± 2.3	0.00	94.7 ± 3.6	0.00
	72 h	NE									

Stop value calculation for control of the pathogen growing over PDA plate in Petri.

## Supplementary Materials

The following are available online at https://www.mdpi.com/2079-6382/8/4/231/s1, Figure S1: Phylogenetic tree from a MP analysis based on the combined genes dataset (ITS, *CAL*, *HIS*, *TEF1*, *TUB*), Table S1: Classification of volatile compounds produced by *Diaporthe apiculatum* strain FPYF 3052, Table S2: The pair primers for amplifying ITS, *CAL, HIS, TEF1*, and *TUB* sequences, Table S3: The reference taxa of *Diaporthe* species with sequences information were downloaded from NCBI nucleotide databases.
