# Supplementary material for: An Endophytic Diaporthe apiculatum Produces Monoterpenes with Inhibitory Activity against Phytopathogenic Fungi
Source: Antibiotics (Basel). 2019 Nov 22;8(4):231. doi: 10.3390/antibiotics8040231 (PMC6963576; doi:10.3390/antibiotics8040231)
Supplement: Supplementary file 1 [file antibiotics-08-00231-s001.zip › antibiotics-608258-supply-/Table S1.docx]

**Table S1.** Classification of volatile compounds produced by *Diaporthe* FPYF3052

| **Monoterpenes**  **(C10H16)** | **Sesquiterpenes**  **(C15H24)** | **Benzenes and Benzene Derivatives** | **Alcohols** | **Hydrocarbons** |
| --- | --- | --- | --- | --- |
| α-thujene | α-muurolene | p-cymene | 2-Cyclohexen-1-ol, 1-methyl-4-(1-methylethyl)-, trans- | 4,5-di-epi-aristolochene |
| β-phellandrene | β-sesquiphellandrene | Biphenylene, 1,2,3,6,7,8,8a,8b-octahydro-4,5-dimethyl- |  |  |
| α-terpinene | (-)-α-himachalene |  |  |  |
| γ-terpinene |  |  |  |  |
| α- terpinolen |  |  |  |  |
| (-)-4-terpineol |  |  |  |  |
