# Supplementary material for: An Endophytic Diaporthe apiculatum Produces Monoterpenes with Inhibitory Activity against Phytopathogenic Fungi
Source: Antibiotics (Basel). 2019 Nov 22;8(4):231. doi: 10.3390/antibiotics8040231 (PMC6963576; doi:10.3390/antibiotics8040231)
Supplement: Supplementary file 1 [file antibiotics-08-00231-s001.zip › antibiotics-608258-supply-/Table S2.docx]

Table S2 Primer sets and corresponding amplification target

| Target gene | Primer | Primer DNA sequence |
| --- | --- | --- |
| CAL | CAL563 | 5'-GACAAATCACCACCAARGAGC-3' |
|  | CL2A | 5'-TTTTTGCATCATGAGTTGGAC-3' |
|  | CL1F | 5'-GARTWCAAGGAGGCCTTCTC-3' |
|  | CL2A | 5'-TTTTTGCATCATGAGTTGGAC-3' |
| HIS | HISdiaF | 5'-GGCTCCCCGYAAGCAGCTCGCCTCC-3' |
|  | HISdiaR | 5'-ATYCCGACTGGATGGTCACACGCTTGG-3' |
| ITS | ITS1 | 5’-TCCGTAGGTGAACCTGCGG-3' |
|  | ITS4 | 5’-TCCTCCGCTTATTGATATGC-3' |
| TEF-1 | EF1-688F | 5'-CGGTCACTTGATCTACAAGTGC-3' |
|  | EF1-1251R | 5'-CCTCGAACTCACCAGTACCG-3' |
| TUB | Bt2a | 5'-GGTAACCAAATCGGTGCTGCTTTC-3' |
|  | Bt2b | 5'-ACCCTCAGTGTAGTGACCCTTGGC-3' |
