# Supplementary material for: An Endophytic Diaporthe apiculatum Produces Monoterpenes with Inhibitory Activity against Phytopathogenic Fungi
Source: Antibiotics (Basel). 2019 Nov 22;8(4):231. doi: 10.3390/antibiotics8040231 (PMC6963576; doi:10.3390/antibiotics8040231)
Supplement: Supplementary file 1 [file antibiotics-08-00231-s001.zip › antibiotics-608258-supply-/Table S3.docx]

**Table S3.**  Reference sequences of *Diaporthe* strains with NCBI access numbers for phylogenetic analysis.

|  | **ITS** | ***TEF1*** | ***TUB*** | ***CAL*** | ***HIS*** |
| --- | --- | --- | --- | --- | --- |
| *Diaporthe acaciigena* CBS 129521 | KC343005 | KC343731 | KC343973 | KC343247 | KC343489 |
| *Diaporthe acerina* CBS 137.27 | KC343006 | KC343732 | KC343974 | KC343248 | KC343490 |
| *Diaporthe alleghaniensis* CBS 495.72 | KC343007 | KC343733 | KC343975 | KC343249 | KC343491 |
| *Diaporthe alnea* CBS 146.46 | KC343008 | KC343734 | KC343976 | KC343250 | KC343492 |
| *Diaporthe alnea* CBS 159.47 | KC343009 | KC343735 | KC343977 | KC343251 | KC343493 |
| *Diaporthe ambigua* CBS 114015 | KC343010 | KC343736 | KC343978 | KC343252 | KC343494 |
| *Diaporthe ambigua* CBS 117167 | KC343011 | KC343737 | KC343979 | KC343253 | KC343495 |
| *Diaporthe ampelina* CBS 111888 | KC343016 | KC343748 | KC343990 | KC343264 | KC343500 |
| *Diaporthe amygdali* CBS 111811 | KC343019 | KC343745 | KC343987 | KC343261 | KC343503 |
| *Diaporthe amygdali* CBS 126679 | KC343022 | KC343742 | KC343984 | KC343258 | KC343506 |
| *Diaporthe anacardii* CBS 720.97 | KC343024 | KC343750 | KC343992 | KC343266 | KC343508 |
| *Diaporthe angelicae* CBS 111592 | KC343027 | KC343753 | KC343995 | KC343269 | KC343511 |
| *Diaporthe angelicae* CBS 123215 | KC343028 | KC343754 | KC343996 | KC343270 | KC343512 |
| *Diaporthe apiculatum* LC3118 | KP267860 | KP267934 | KP293440 | - | KP293514 |
| *Diaporthe apiculatum* LC3418 | KP267896 | KP267970 | KP293476 | - | - |
| *Diaporthe arecae* CBS 161.64 | KC343032 | KC343758 | KC344000 | KC343274 | KC343516 |
| *Diaporthe arecae* CBS 535.75 | KC343033 | KC343759 | KC344001 | KC343275 | KC343517 |
| *Diaporthe arengae* CBS 114979 | KC343034 | KC343760 | KC344002 | KC343276 | KC343518 |
| *Diaporthe aspalathi* CBS 117168 | KC343035 | KC343761 | KC344003 | KC343277 | KC343519 |
| *Diaporthe aspalathi* CBS 117169 | KC343036 | KC343762 | KC344004 | KC343278 | KC343520 |
| *Diaporthe australafricana* CBS 111886 | KC343038 | KC343764 | KC344006 | KC343280 | KC343522 |
| *Diaporthe australafricana* CBS 113487 | KC343039 | KC343765 | KC344007 | KC343281 | KC343523 |
| *Diaporthe batatas* CBS 122.21 | KC343040 | KC343766 | KC344008 | KC343282 | KC343524 |
| *Diaporthe beckhausii* CBS 138.27 | KC343041 | KC343767 | KC344009 | KC343283 | KC343525 |
| *Diaporthe bicincta* CBS 121004 | KC343134 | KC343860 | KC344102 | KC343376 | KC343618 |
| *Diaporthe brasiliensis* CBS 133183 | KC343042 | KC343768 | KC344010 | KC343284 | KC343526 |
| *Diaporthe brasiliensis* LGMF926 | KC343043 | KC343769 | KC344011 | KC343285 | KC343527 |
| *Diaporthe carpini* CBS 114437 | KC343044 | KC343770 | KC344012 | KC343286 | KC343528 |
| *Diaporthe caulivora* CBS 127268 | KC343045 | KC343771 | KC344013 | KC343287 | KC343529 |
| *Diaporthe caulivora* CBS 178.55 | KC343046 | KC343772 | KC344014 | KC343288 | KC343530 |
| *Diaporthe celastrina* CBS 139.27 | KC343047 | KC343773 | KC344015 | KC343289 | KC343531 |
| *Diaporthe* cf. *heveae* 1 RG-2013 CBS 852.97 | KC343116 | KC343971 | KC344213 | KC343487 | KC343729 |
| *Diaporthe* cf. *heveae* 2 RG-2013 CBS 681.84 | KC343117 | KC343842 | KC344084 | KC343358 | KC343600 |
| *Diaporthe* cf. *nobilis* RG-2013 CBS 113470 | KC343146 | KC343872 | KC344114 | KC343388 | KC343630 |
| *Diaporthe* cf. *nobilis* RG-2013 CBS 116953 | KC343147 | KC343873 | KC344115 | KC343389 | KC343631 |
| *Diaporthe* cf. *nobilis* RG-2013 CBS 200.39 | KC343151 | KC343877 | KC344119 | KC343393 | KC343635 |
| *Diaporthe chamaeropis* CBS 454.81 | KC343048 | KC343774 | KC344016 | KC343290 | KC343532 |
| *Diaporthe chamaeropis* CBS 753.70 | KC343049 | KC343775 | KC344017 | KC343291 | KC343533 |
| *Diaporthe charlesworthii* BRIP 54884m | KJ197288 | KJ197250 | KJ197268 | - | - |
| *Diaporthe cinerascens* CBS 719.96 | KC343050 | KC343776 | KC344018 | KC343292 | KC343534 |
| *Diaporthe citri* CBS 199.39 | KC343051 | KC343777 | KC344019 | KC343293 | KC343535 |
| *Diaporthe citri* CBS 230.52 | KC343052 | KC343778 | KC344020 | KC343294 | KC343536 |
| *Diaporthe convolvuli* CBS 124654 | KC343054 | KC343780 | KC344022 | KC343296 | KC343538 |
| *Diaporthe crataegi* CBS 114435 | KC343055 | KC343781 | KC344023 | KC343297 | KC343539 |
| *Diaporthe crotalariae* CBS 162.33 | KC343056 | KC343782 | KC344024 | KC343298 | KC343540 |
| *Diaporthe cucurbitae* CBS 136.25 | KC343031 | KC343757 | KC343999 | KC343273 | KC343515 |
| *Diaporthe cuppatea* CBS 117499 | KC343057 | KC343783 | KC344025 | KC343299 | KC343541 |
| *Diaporthe cynaroidis* CBS 122676 | KC343058 | KC343784 | KC344026 | KC343300 | KC343542 |
| *Diaporthe decedens* CBS 109772 | KC343059 | KC343785 | KC344027 | KC343301 | KC343543 |
| *Diaporthe decedens* CBS 114281 | KC343060 | KC343786 | KC344028 | KC343302 | KC343544 |
| *Diaporthe detrusa* CBS 109770 | KC343061 | KC343787 | KC344029 | KC343303 | KC343545 |
| *Diaporthe detrusa* CBS 114652 | KC343062 | KC343788 | KC344030 | KC343304 | KC343546 |
| *Diaporthe elaeagni* CBS 504.72 | KC343064 | KC343790 | KC344032 | KC343306 | KC343548 |
| *Diaporthe endophytica* CBS 133811 | KC343065 | KC343791 | KC344033 | KC343307 | KC343549 |
| *Diaporthe endophytica* LGMF928 | KC343068 | KC343794 | KC344036 | KC343310 | KC343552 |
| *Diaporthe eres* CBS 101742 | KC343073 | KC343799 | KC344041 | KC343315 | KC343557 |
| *Diaporthe eres* CBS 109767 | KC343075 | KC343801 | KC344043 | KC343317 | KC343559 |
| *Diaporthe eres* CBS 439.82 | KC343090 | KC343816 | KC344058 | KC343332 | KC343574 |
| *Diaporthe eugeniae* CBS 444.82 | KC343098 | KC343824 | KC344066 | KC343340 | KC343582 |
| *Diaporthe fibrosa* CBS 109751 | KC343099 | KC343825 | KC344067 | KC343341 | KC343583 |
| *Diaporthe fibrosa* CBS 113830 | KC343100 | KC343826 | KC344068 | KC343342 | KC343584 |
| *Diaporthe foeniculacea* CBS 111553 | KC343101 | KC343827 | KC344069 | KC343349 | KC343585 |
| *Diaporthe foeniculacea* CBS 123208 | KC343104 | KC343830 | KC344072 | KC343346 | KC343588 |
| *Diaporthe foeniculacea* CBS 187.27 | KC343107 | KC343833 | KC344075 | KC343343 | KC343591 |
| *Diaporthe ganjae* CBS 180.91 | KC343112 | KC343838 | KC344080 | KC343354 | KC343596 |
| *Diaporthe gardeniae* CBS 288.56 | KC343113 | KC343839 | KC344081 | KC343355 | KC343597 |
| *Diaporthe helianthi* CBS 344.94 | KC343114 | KC343840 | KC344082 | KC343356 | KC343598 |
| *Diaporthe helianthi* CBS 592.81 | KC343115 | KC343841 | KC344083 | KC343357 | KC343599 |
| *Diaporthe hickoriae* CBS 145.26 | KC343118 | KC343844 | KC344086 | KC343360 | KC343602 |
| *Diaporthe hongkongensis* CBS 115448 | KC343119 | KC343845 | KC344087 | KC343361 | KC343603 |
| *Diaporthe hordei* CBS 481.92 | KC343120 | KC343846 | KC344088 | KC343362 | KC343604 |
| *Diaporthe impulsa* CBS 114434 | KC343121 | KC343847 | KC344089 | KC343363 | KC343605 |
| *Diaporthe impulsa* CBS 141.27 | KC343122 | KC343848 | KC344090 | KC343364 | KC343606 |
| *Diaporthe inconspicua* CBS 133813 | KC343123 | KC343850 | KC344092 | KC343366 | KC343608 |
| *Diaporthe inconspicua* LGMF922 | KC343124 | KC343849 | KC344091 | KC343365 | KC343607 |
| *Diaporthe infecunda* CBS 133812 | KC343126 | KC343852 | KC344094 | KC343368 | KC343610 |
| *Diaporthe infecunda* LGMF933 | KC343132 | KC343858 | KC344100 | KC343374 | KC343616 |
| *Diaporthe longicolla* PL4 | KC344167 | KC343441 | KC343683 | - | - |
| *Diaporthe longispora* CBS 194.36 | KC343135 | KC343861 | KC344103 | KC343377 | KC343619 |
| *Diaporthe lusitanicae* CBS 123212 | KC343136 | KC343862 | KC344104 | KC343378 | KC343620 |
| *Diaporthe lusitanicae* CBS 123213 | KC343137 | KC343863 | KC344105 | KC343379 | KC343621 |
| *Diaporthe manihotia* CBS 505.76 | KC343138 | KC343864 | KC344106 | KC343380 | KC343622 |
| *Diaporthe mayteni* CBS 133185 | KC343139 | KC343865 | KC344107 | KC343381 | KC343623 |
| *Diaporthe megalospora* CBS 143.27 | KC343140 | KC343866 | KC344108 | KC343383 | KC343624 |
| *Diaporthe melonis* CBS 435.87 | KC343141 | KC343867 | KC344109 | KC343382 | KC343625 |
| *Diaporthe melonis* CBS 507.78 | KC343142 | KC343868 | KC344110 | KC343384 | KC343626 |
| *Diaporthe musigena* CBS 129519 | KC343143 | KC343869 | KC344111 | KC343385 | KC343627 |
| *Diaporthe neilliae* CBS 144.27 | KC343144 | KC343870 | KC344112 | KC343386 | KC343628 |
| *Diaporthe neoarctii* CBS 109490 | KC343145 | KC343871 | KC344113 | KC343387 | KC343629 |
| *Diaporthe nomurai* CBS 157.29 | KC343154 | KC343880 | KC344122 | KC343396 | KC343638 |
| *Diaporthe novem* CBS 127270 | KC343156 | KC343882 | KC344124 | KC343398 | KC343640 |
| *Diaporthe novem* CBS 354.71 | KC343158 | KC343884 | KC344126 | KC343400 | KC343642 |
| *Diaporthe oncostoma* CBS 100454 | KC343160 | KC343886 | KC344128 | KC343402 | KC343644 |
| *Diaporthe oncostoma* CBS 109741 | KC343161 | KC343887 | KC344129 | KC343403 | KC343645 |
| *Diaporthe oxe* CBS 133186 | KC343164 | KC343890 | KC344132 | KC343406 | KC343648 |
| *Diaporthe oxe* CBS 133187 | KC343165 | KC343891 | KC344133 | KC343407 | KC343649 |
| *Diaporthe padi* var. *padi* CBS 114200 | KC343169 | KC343895 | KC344137 | KC343411 | KC343653 |
| *Diaporthe padi* var. *padi* CBS 114649 | KC343170 | KC343896 | KC344138 | KC343412 | KC343654 |
| *Diaporthe paranensis* CBS 133184 | KC343171 | KC343897 | KC344139 | KC343413 | KC343655 |
| *Diaporthe penetriteum* LC3215 | KP267879 | KP267953 | KP293459 | - | KP293532 |
| *Diaporthe perjuncta* CBS 109745 | KC343172 | KC343898 | KC344140 | KC343414 | KC343656 |
| *Diaporthe perseae* CBS 151.73 | KC343173 | KC343899 | KC344141 | KC343415 | KC343657 |
| *Diaporthe phaseolorum* CBS 116019 | KC343175 | KC343901 | KC344143 | KC343417 | KC343659 |
| *Diaporthe phaseolorum* CBS 116020 | KC343176 | KC343902 | KC344144 | KC343418 | KC343660 |
| *Diaporthe pseudomangiferae* CBS 101339 | KC343181 | KC343907 | KC344149 | KC343423 | KC343665 |
| *Diaporthe pseudomangiferae* CBS 388.89 | KC343182 | KC343908 | KC344150 | KC343424 | KC343666 |
| *Diaporthe pseudophoenicicola* CBS 176.77 | KC343183 | KC343909 | KC344151 | KC343425 | KC343667 |
| *Diaporthe pseudophoenicicola* CBS 462.69 | KC343184 | KC343910 | KC344152 | KC343426 | KC343668 |
| *Diaporthe pustulata* CBS 109742 | KC343185 | KC343911 | KC344153 | KC343427 | KC343669 |
| *Diaporthe pustulata* CBS 109784 | KC343187 | KC343913 | KC344155 | KC343429 | KC343671 |
| *Diaporthe raonikayaporum* CBS 133182 | KC343188 | KC343914 | KC344156 | KC343430 | KC343672 |
| *Diaporthe rhoina* CBS 146.27 | KC343189 | KC343915 | KC344157 | KC343431 | KC343673 |
| *Diaporthe rudis* CBS 109768 | KC343233 | KC343960 | KC344202 | KC343476 | KC343718 |
| *Diaporthe rudis* CBS 113201 | KC343234 | KC343955 | KC344197 | KC343471 | KC343713 |
| *Diaporthe saccarata* CBS 116311 | KC343190 | KC343916 | KC344158 | KC343432 | KC343674 |
| *Diaporthe schini* CBS 133181 | KC343191 | KC343917 | KC344159 | KC343433 | KC343675 |
| *Diaporthe schini* LGMF910 | KC343192 | KC343918 | KC344160 | KC343434 | KC343676 |
| *Diaporthe sclerotioides* CBS 296.67 | KC343193 | KC343919 | KC344161 | KC343435 | KC343677 |
| *Diaporthe sclerotioides* CBS 710.76 | KC343194 | KC343920 | KC344162 | KC343436 | KC343678 |
| *Diaporthe scobina* CBS 251.38 | KC343195 | KC343921 | KC344163 | KC343437 | KC343679 |
| *Diaporthe sennicola* CFCC 51634 | KY203722 | KY228883 | KY228889 | KY228873 | KY228879 |
| *Diaporthe sojae* CBS 100.87 | KC343196 | KC343922 | KC344164 | KC343438 | KC343680 |
| *Diaporthe sojae* CBS 116017 | KC343197 | KC343923 | KC344165 | KC343439 | KC343681 |
| *Diaporthe sojae* CBS 180.55 | KC343200 | KC343926 | KC344168 | KC343442 | KC343684 |
| *Diaporthe* sp. 1 RG-2013 CBS 119639 | KC343202 | KC343843 | KC344085 | KC343359 | KC343601 |
| *Diaporthe* sp. 1 RG-2013 LGMF947 | KC343203 | KC343928 | KC344170 | KC343444 | KC343686 |
| *Diaporthe* sp. 2 RG-2013 LGMF932 | KC343204 | KC343929 | KC344171 | KC343445 | KC343687 |
| *Diaporthe* sp. 3 RG-2013 CBS 287.29 | KC343205 | KC343930 | KC344172 | KC343446 | KC343688 |
| *Diaporthe* sp. 4 RG-2013 LGMF944 | KC343206 | KC343931 | KC344173 | KC343448 | KC343689 |
| *Diaporthe* sp. 5 RG-2013 CBS 125575 | KC343207 | KC343932 | KC344174 | KC343447 | KC343690 |
| *Diaporthe* sp. 6 RG-2013 CBS 115584 | KC343208 | KC343933 | KC344175 | KC343449 | KC343691 |
| *Diaporthe* sp. 6 RG-2013 CBS 115595 | KC343209 | KC343934 | KC344176 | KC343450 | KC343692 |
| *Diaporthe* sp. 7 RG-2013 CBS 458.78 | KC343210 | KC343935 | KC344177 | KC343451 | KC343693 |
| *Diaporthe* sp. 8 RG-2013 LGMF925 | KC343211 | KC343936 | KC344178 | KC343452 | KC343694 |
| *Diaporthe stictica* CBS 370.54 | KC343212 | KC343730 | KC343972 | KC343246 | KC343488 |
| *Diaporthe subclavata* ZJUD95 | KJ490630 | KJ490509 | KJ490451 | - | - |
| *Diaporthe subordinaria* CBS 101711 | KC343213 | KC343938 | KC344180 | KC343454 | KC343696 |
| *Diaporthe subordinaria* CBS 464.90 | KC343214 | KC343939 | KC344181 | KC343455 | KC343697 |
| *Diaporthe tecomae* CBS 100547 | KC343215 | KC343940 | KC344182 | KC343456 | KC343698 |
| *Diaporthe terebinthifolii* CBS 133180 | KC343216 | KC343941 | KC344184 | KC343457 | KC343699 |
| *Diaporthe terebinthifolii* LGMF907 | KC343217 | KC343942 | KC344183 | KC343458 | KC343700 |
| *Diaporthe toxica* CBS 534.93 | KC343220 | KC343943 | KC344185 | KC343459 | KC343701 |
| *Diaporthe toxica* CBS 535.93 | KC343221 | KC343946 | KC344188 | KC343462 | KC343704 |
| *Diaporthe vaccinii* CBS 122112 | KC343224 | KC343954 | KC344196 | KC343470 | KC343712 |
| *Diaporthe vaccinii* CBS 160.32 | KC343228 | KC343947 | KC344189 | KC343463 | KC343705 |
| *Diaporthe vexans* CBS 127.14 | KC343229 | KC343950 | KC344192 | KC343466 | KC343708 |
| *Diaporthe woodii* CBS 558.93 | KC343244 | KC343959 | KC344201 | KC343475 | KC343717 |
| *Diaporthe woolworthii* CBS 148.27 | KC343245 | KC343970 | KC344212 | KC343486 | KC343728 |
| *Diaporthella corylina* CBS 121124 | KC343004 | KC343937 | KC344179 | KC343453 | KC343695 |
