# Supplementary figures and images for: An Endophytic Diaporthe apiculatum Produces Monoterpenes with Inhibitory Activity against Phytopathogenic Fungi
Source: Antibiotics (Basel). 2019 Nov 22;8(4):231. doi: 10.3390/antibiotics8040231 (PMC6963576; doi:10.3390/antibiotics8040231)

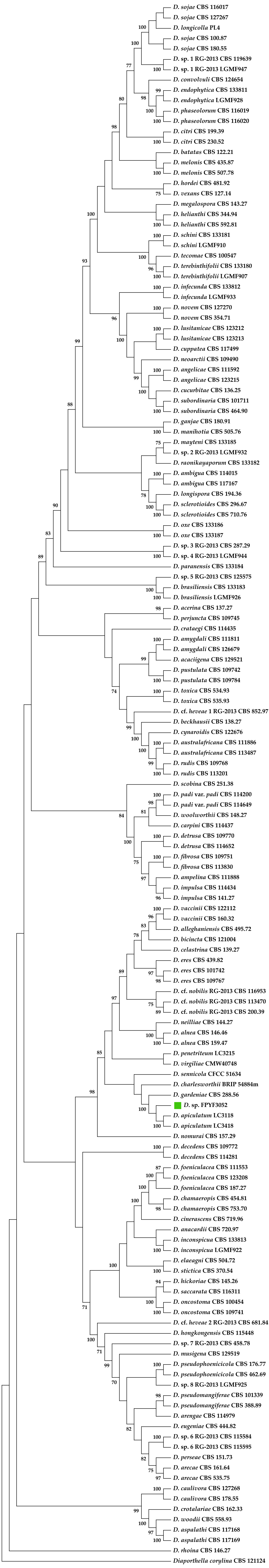

Supplement: Supplementary file 1 [file antibiotics-08-00231-s001.zip › antibiotics-608258-supply-/Figure S1.pdf]
